# Trajectories of Hospitalization in COVID-19 Patients: An Observational Study in France

**DOI:** 10.3390/jcm9103148

**Published:** 2020-09-29

**Authors:** Pierre-Yves Boëlle, Tristan Delory, Xavier Maynadier, Cécile Janssen, Renaud Piarroux, Marie Pichenot, Xavier Lemaire, Nicolas Baclet, Pierre Weyrich, Hugues Melliez, Agnès Meybeck, Jean-Philippe Lanoix, Olivier Robineau

**Affiliations:** 1 Sorbonne Université, Institut Pierre Louis d’Epidémiologie et de Santé Publique, INSERM, Assistance Publique – Hôpitaux de Paris, 75012 Paris, France; tdelory@ch-annecygenevois.fr (T.D.); xav_m@hotmail.fr (X.M.); renaud.piarroux@aphp.fr (R.P.); 2 Centre Hospitalier Annecy Genevois, 74370 Epagny–Metz-Tessy, France; cjanssen@ch-annecygenevois.fr; 3 Centre Hospitalier Victor Provot, 59100 Roubaix, France; marie.pichenot@ch-roubaix.fr; 4 Service Maladies infectieuses, Centre Hospitalier de Douai, 59500 Douai, France; xavier.Lemaire@ch-douai.fr; 5 Department of Infectious Diseases, Lille Catholic Hospitals, F-59160 Lille, France; Baclet.Nicolas@ghicl.net (N.B.); Weyrich.Pierre@ghicl.net (P.W.); 6 Service de médecine interne, Hôpital de la région de Saint-Omer, 62570 Helfaut, France; hugues.melliez@ch-stomer.fr; 7 Service Universitaire des maladies Infectieuses et du Voyageur, 59200 Tourcoing, France; ameybeck@ch-tourcoing.fr; 8 Service de Maladies Infectieuses et tropicales, CHU Amiens-Picardie, 80000 Amiens, France; Lanoix.Jean-Philippe@chu-amiens.fr; 9 AGIR UR UPJV 4294, CURS, Université Picardie Jules Verne, 80000 Amiens, France

**Keywords:** COVID-19, hospital trajectories, mixture model, hospital mortality, length of stay, ICU

## Abstract

Describing the characteristics of COVID-19 patients in the hospital is of importance to assist in the management of hospital capacity in the future. Here, we analyze the trajectories of 1321 patients admitted to hospitals in northern and eastern France. We found that the time from onset to hospitalization decreased with age, from 7.3 days in the 20–65 year-olds to 4.5 in the >80 year-olds (*p* < 0.0001). Overall, the length of stay in the hospital was 15.9 days, and the death rate was 20%. One patient out of four was admitted to the intensive care unit (ICU) for approximately one month. The characteristics of trajectories changed with age: fewer older patients were admitted to the ICU and the death rate was larger in the elderly. Admission shortly after onset was associated with increased mortality (odds-ratio (OR) = 1.8, Confidence Interval (CI) 95% [1.3, 2.6]) as well as male sex (OR = 2.1, CI 95% [1.5, 2.9]). Time from admission within the hospital to the transfer to ICU was short. The age- and sex-adjusted mortality rate decreased over the course of the epidemic, suggesting improvement in care over time. In the SARS-CoV-2 epidemic, the urgent need for ICU at admission and the prolonged length of stay in ICU are a challenge for bed management and organization of care.

## 1. Introduction

COVID-19 was first detected in the eastern part of France in January 2020 in travelers from China [1]. After an initial period where the control of local clusters was successful, increased community circulation was noted by late February, especially in the northern and eastern parts of France [2]. The number of cases requiring intensive care increased from early March, leading to Intensive Care Units (ICU) overcrowding and the decision of a national lockdown on March 17th to control the epidemic.

During the COVID-19 first wave in France, the hospital system underwent a dynamical reorganization to accommodate more patients in both intensive care and conventional hospitalization, with a doubling of the number of ICU beds. Characterizing patients’ trajectories in the hospital, especially the length of stay in different wards, is required to understand why and how the system had to change. This is necessary to plan for future demand in beds as renewed increase in circulation is occurring in many countries. Furthermore, it is also required to appreciate whether COVID-19 care improved over time, investigating mortality as well as admission to the ICU or length of stay to examine future capacity requirements more precisely. Information on the time between disease onset and admission to the hospital or to the intensive care unit (ICU) is also scarce in the literature and may be of use to help define best care.

Yet, answering these questions is challenging. This is because the analysis must be done as some patients are still hospitalized and their total duration in the hospital and final outcome are unknown. Second, the characteristics of hospitalized patients changed over time, as a result of both the dynamics of the disease and, later, the lockdown. Dedicated statistical procedures are required to account for unknown outcome or censoring in the data. Adjustment for confusion factors in estimating improvement in outcomes for COVID-19 patients, as well as in estimates on the sojourn durations in medical and ICU wards, is also needed.

Here, we analyzed the patients hospitalized in 7 French hospitals in the regions where the epidemic was very active in France (East and North). We analyzed individual characteristics linked to specific trajectories and final outcomes using a mixture model.

## 2. Experimental Section

### 2.1. Data

Seven general hospitals participated in the study. All but one of these hospitals were from northern France and covered a large part of the local population. The last hospital was from eastern France, in the location where the first clusters of COVID-19 cases occurred. All patients aged ≥ 18 years old admitted for COVID-19 in one of the participating hospitals between 1 January and 31 May were included. COVID-19 diagnosis was based on positive Polymerase Chain Reaction (PCR) test or evocative chest scan. For all patients, we obtained demographic data, date of symptom onset, date of hospitalization, and dates of transfer between wards (ICU or not). We also obtained dates and status at discharge or at the end of data collection. This multicentric observational study was registered to the French authorities under the numbers MR004-202004-006 (Annecy Genevois hospital) and MR04-2020-03 (Tourcoing hospital), using the MR-004 referral methodology of the “Commission Nationale de l’Informatique et des Libertés”. Data transfer from hospitals to Institut Pierre Louis d’Epidémiologie et de Santé Publique was ruled by a transfer agreement between the three parties under the reference C20/0506. The data collection process was in line with the European General Data Protection Regulation (GDPR). 

### 2.2. Statistical Analysis

Patients with COVID-19 admitted to the hospital ultimately experienced one of two competing outcomes, death or discharge. Analyzing data on patient outcome and hospital trajectories during an epidemic is difficult because patients kept being admitted over the whole period and the hospital stay was long. Thus, some patients were still hospitalized at the time of data analysis. This leads to censoring in the data, as the ultimate event is unknown. Time-to-event analysis allows taking this into account. But the analysis of time spent in the hospital or in the ICU for an acute event such as COVID-19 needs a different perspective than usual time-to-event analysis, in which duration to the event defines the outcome. Indeed, the length of the hospital stay does not define a good or a bad outcome [3]. For COVID-19, the main point of interest is the proportion of patients experiencing each outcome. The time to reach the outcome is secondary but is necessary for capacity planning. Thus, time from disease onset to hospitalization was analyzed accounting for truncation (see Statistical Methods in Supplementary Information). As initial inspection suggested a bimodal distribution, we fitted a mixture of 2 distributions to the data: an exponential distribution to capture “quick progressors” and a log-normal distribution for “slow progressors”. This analysis was made with proportions of quick/slow progressors according to age. Age was categorized into three category (18–65, 66–80, >80 years) for balance between groups and because it corresponds with retirement (65 years old) and increased triage before ICU admission (80 years old).

For some patients, the final outcome, after hospital admission, was still unknown as they remained hospitalized. We, therefore, used parametric survival modelling to account for censoring of future events. An additional issue is that 2 outcomes are possible, death or discharge. We, therefore, fitted a mixture of parametric time-to-event distributions. In this approach, the total percentage of patients reaching each outcome is estimated independently from the time it takes to reach that outcome. We defined a trajectory as the sequence and timing of events from disease onset to final outcome. Before admission, we used the “quick” or “slow” progressors described above. After hospital admission, we described trajectories with 3 possible paths: the first was the admission to intensive care, the second a stay in conventional care ending in death, and the third ending in discharge. Then, for patients who entered the ICU, we modelled two further paths: death or discharge to conventional care. Patients who died in the week following discharge from the ICU were counted as dead in the ICU. When there was more than one ICU stay, the patient trajectory was simplified to a single stay in the ICU from the first entry to the last discharge from the ICU. Patients still hospitalized at the time of the study were right-censored according to the stage they had reached (ICU or hospitalization). We used log-normal distributions for the time to the event and estimated mean duration of stay in the successive wards from the fitted distributions, with a sensitivity analysis using exponential distributions. For the main analysis, we fixed the coefficient of variation for each outcome in each age class. Confidence intervals were determined using normal approximations. 

We also analyzed the overall outcome of patients, death or discharge, without accounting for path details. We examined the effect of the date of admission using a 1-week sliding window to smooth estimates. We modelled the percentage of death after admission according to age, and computed age-adjusted odds ratios for death according to sex and duration from onset to hospital admission (see Statistical Methods in the Supplementary Information for more detail). Computations were done using R (v3.6. R Foundation for Statistical Computing, Vienna, Austria).

## 3. Results

**Figure 1:**
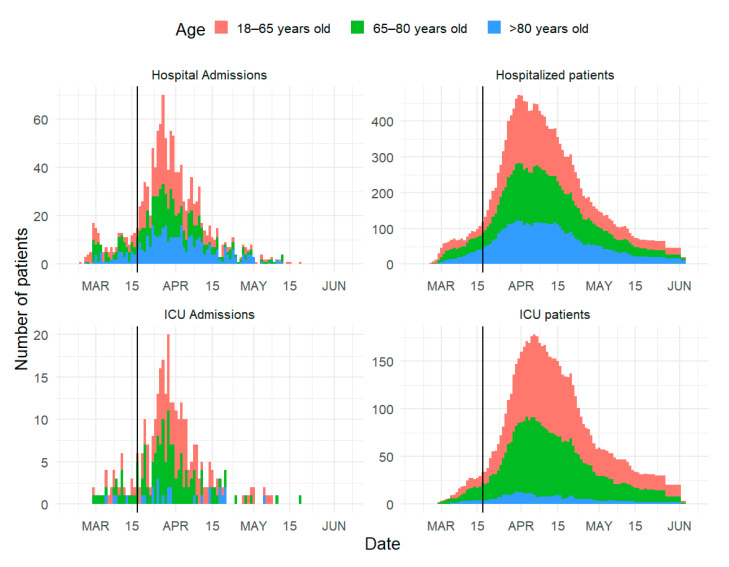
Daily admissions in the hospital (**top left**) and the ICU (**bottom left**) and overall occupation in the hospital (**top right**) and the ICU (**bottom right**) during the COVID-19 epidemic. The vertical black line is the date of the lockdown (17 March). ICU=Intensive Care Unit.

There were 1321 patients admitted to the participating hospitals for COVID-19 between 1 January and 31 May 2020 (Table 1). The first patient was admitted on 24 February, and the last on 19 May. The hospital admission curve peaked on 27 March, 10 days after the national lockdown, with 70 daily admissions (Figure 1). The peak in admission to the ICU was 2 days later, on 29 March, with 20 admissions. The maximum number of beds occupied in the hospital was 473 over the period, of which 178 beds (38%) were in intensive care. During this period, the number of ICU beds for COVID-19 patients exceeded the usual medical ICU capacity by 120%. Approximately one third of the patients were more than 80 years old, and 40% were less than 65 years old. There were slightly more men than women, except in the oldest age class.

**Figure 2:**
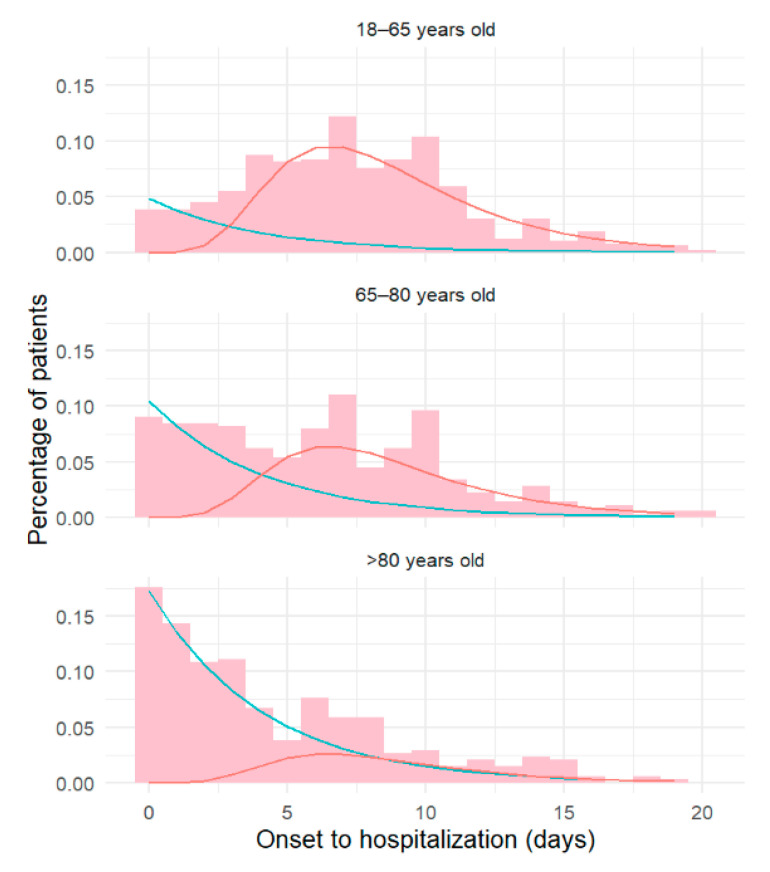
Time from onset to hospitalization according to age. The lines correspond to the two components of the mixture analysis: slow progressor (red), rapid progressor (blue). Histogram is shown in pink.

The mean time from onset to hospitalization decreased with age, from 7.3 days (+/−0.2 standard deviation, SD) in the ≤65 year-olds, to 6.2 days (+/−0.2) in the 65–80 and to 4.5 days (+/−0.2) in the >80 year-olds (*p* < 0.0001). The overall distribution was bimodal, with a first peak shortly after onset and a second peak in the second week after onset. The mixture analysis yielded the superposition of a “quick” exponential distribution with mean 4.1 days (+/−2) and a “slow” log-normal distribution with mean 9.2 days (+/−1.7). The proportion of “quick” progressors increased with age, from 26% in the ≤65 year-olds, to 44% in the 65–80 year-olds, and 90% in the >80 year-olds (Figure 2).

**Figure 3:**
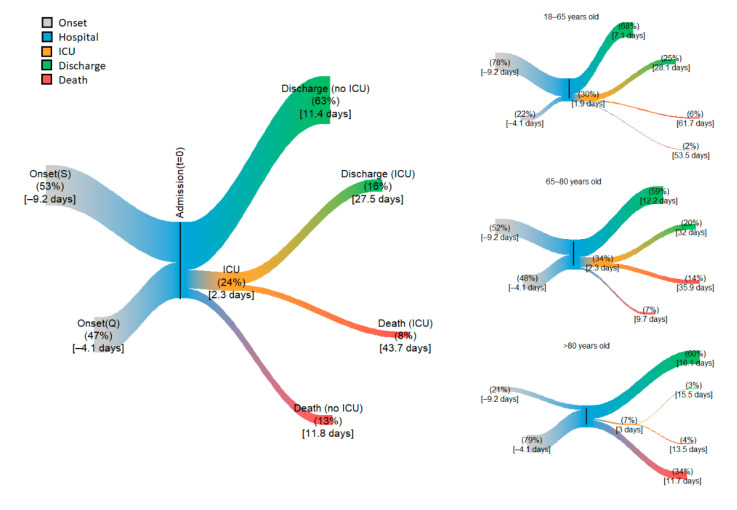
Summary of patient trajectories from onset to admission. Time flows from left to right. Onset (S) are slow progressors, Onset (Q) are quick progressors. The width of the branches is proportional to the percentage of patients. Branches end at the average length of stay in each ward. Left side: All patients, right side, top to bottom, age groups: 18–65 years old, 65–80 years old, >80 years old.

The characteristics of patients’ trajectories are summarized in Figure 3 and described with more detail in Table 2. Overall, patients stayed in the hospital for about 2 weeks. One patient out of 5 (20%) died after an average of 20 days in the hospital. During the course of the hospital stay, 1 patient out of 4 (25%) was admitted to the ICU shortly after admission within a span of 2 days on average. Admission in ICU was on the day of hospital admission for the majority of the patients (58%). The average time requiring intensive care was one month. The average length of stay in ICU was of 27 days +/− SD for those who survived (two-thirds) and of 45 days +/− SD for those who died (one-third). In the patients who did not go to the ICU, the length of stay was shorter overall (11 days +/− SD), with little difference between those discharged or those who died. The results were similar using exponential distributions, although durations were a bit shorter (see Supplementary Tables S1 and S2).

The characteristics of these trajectories showed two marked differences with age. Older patients (66–80 and >80-year-old) were less likely to be admitted in ICU and had a higher mortality (Figure 3 and Table 2). More precisely, overall mortality increased with age from 9% in the ≤65-year-old to 38% in the >80-year-old patients, while ICU admission respectively decreased from 30% to 7%. The overall length of stay was also affected by age, with shorter stays for older patients who died but longer stays when they were discharged alive. The converse was observed in the younger patients (≤65-year-old).

The overall risk of death increased with age (Table 2) with OR = 2.5 (CI 95% [1.3, 4.9]) for the 65–80 years old, and OR = 6.4 (CI 95% [3.4, 12.2]) for the >80 years old, relative to the less than 65 years old. In addition to age, we found that risk factors for death included hospitalization in the 5 days after onset rather than later, irrespective of age (adusted odds-ratio, aOR = 1.8, CI 95% [1.3, 2.6], adjusted for age) and being a male patient (aOR = 2.1, CI 95% [1.5, 2.9], adjusted for age).

**Figure 4:**
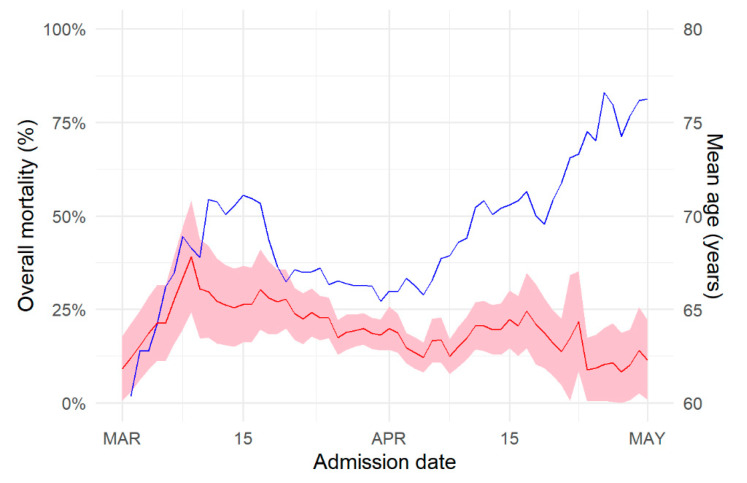
Mortality rate (red) and mean age (blue) at date of hospital admission. Mortality rate was corrected for censoring (shaded area is 95% confidence interval).

Figure 4 shows that expected mortality in newly admitted patients decreased over time. The raw odds ratios for mortality, relative to the period ending 15 March, were 0.78 [0.5, 1.2] from 15 March to 1 April, 0.71 [0.4, 1.1] from 1 April to 15 April, and 0.70 [0.4, 1.3] afterwards. Yet, the mean age of patients at admission increased at first during the course of the outbreak, reaching 70 years old at the time of the lockdown, decreased down to 63 years during the lockdown, at the time of the epidemic peak, to increase again afterward. To disentangle age effects form that of reduction in mortality over time, we computed age-adjusted odds ratios of death over time. Relative to the period ending on March 15, these were 0.79 [0.5, 1.3] from 15 March to 1 April, 0.57 [0.33, 0.97] from 1 April to 15 April, and 0.36 [0.18, 0.72] afterwards, showing larger reduction in mortality once changes in age were taken into account. The median time spent in the hospital was similar over the first three periods and increased in the last fortnight (Table 3). This change was mostly driven by longer time to discharge.

## 4. Discussion

This work highlights that trajectories and outcomes of hospitalized COVID-19 patients followed different patterns depending on age and time between onset and hospitalization. It shows that hospitalization in ICU happens rapidly after admission in the hospital and that overall hospital stays were long.

Patient trajectories began with time from onset to admission: some patients progressed rapidly and required hospitalization in the week after the onset of symptoms (quick progressors), while others arrived at the hospital after a longer time since the onset of symptoms (slow progressors). Bimodal distributions in trajectories of COVID-19 patients have already been reported; in an early case series, an old patient died rapidly after disease onset, and two young men had worsening 10 days after onset [4]. A case series from New York also reported a bimodal distribution from disease onset to intubation in the first few days, then around 9 days [5]. We found that this bimodal distribution is informed by age: young individuals were on average hospitalized later than older patients. It has been proposed that COVID-19 causes an early disease due to the acute viral infection, and a later disease related to the inflammatory reaction. This later phase of clinical worsening leads to increased disease severity [6,7]. Along with this description, older patients may be more prone to the first type of disease and younger patients to the second type. However, it is also possible that the disease is recognized later in the old, because of the typical low symptomatology and non-prodromal nature of viral infections in the elderly [8]. Furthermore, atypical symptoms have been described in the elderly that may complicate the initial diagnosis of COVID-19. Thus, especially in the elderly, a shorter duration between the onset and admission to hospital/ICU could be related to a measurement bias. From a practical point of view, short duration from the onset should be considered as an additional risk factor in hospitalized patients. Reinforced clinical vigilance in the elderly is required as soon as the first symptoms appear in order to plan hospitalization as soon as possible to allow optimal treatment. Although no etiological treatment for COVID-19 has been discovered, treatments such as anticoagulation or corticosteroids can improve the outcome of patients [9,10,11]. Starting these therapies as early as possible could further reduce mortality.

During the course of the disease, we found that approximately one patient out of four required intensive care. The time to ICU admission was short, in the two days following admission for the most part. This highlights again that the disease can progress rapidly, with major consequences in hospital organization. Knowing that a fourth of patients will be transferred in ICU, and the majority during the first three days, since hospital admission is of key importance for bed management in ICUs. Furthermore, starting treatments as soon as possible after admission to the hospital might avoid clinical aggravation and transfer to the ICU [12]. In severe pneumonia, late admission to ICU is associated with poor outcome [13]. However, admission rate in ICU due to COVID-19 might be lower in the near future as the use of effective therapy such as steroids might be generalized [11]. Thus, the management of hospitalized patients may need to be reassessed to ensure early and appropriate care to all patients. A striking feature was that old patients were admitted less frequently to the ICU than others. Criteria for admission to the ICU may change during a pandemic situation, to favor younger patients when the bed capacity in ICU is low [14]. However, older patients were also less frequently admitted to the ICU during the 2009 influenza pandemic [15], when shortage of ICU beds was not an issue. This suggests that the participating hospitals did not significantly alter the rules of triage for admission to the ICU during the course of the outbreak.

A recent systematic review evaluating the length of stay (LOS) in hospital and/or ICU for patients with COVID-19 showed wide variation in the average LOS, ranging from 4 to 53 days depending on country, admission criteria, and timing of the pandemic [16]. Yet, most surveys included in this review concerned small number of subjects who were hospitalized during the first month of the epidemic and did not account for censoring. Here, we had little censoring (5%) and used dedicated statistical procedures. We found that the length of stay in the hospital was highly variable, depending on age and wards (ICU or not). In most cases, an extended period of stay was found in the young patients, especially in those with poor outcomes. The durations of ICU stays were long in the young patients compared to other pulmonary diseases requiring intensive care. For example, the median LOS in ICU for all-cause acute respiratory distress syndrome was 18 days in a recent trial, while it was 32 days in the younger age group in our study [17]. The extent of initial lung damage may contribute to this extended duration, but also to the high frequency of the need for another organ-supportive care, such as dialysis [18]. The lack of downstream beds for the management of COVID-19 patients requiring extensive post-intensive care is also a possibility that may explain the long duration of hospitalization in ICU for surviving patients. Managing healthcare capacity is of prime importance during a health disaster. While most research focused on ICU and conventional units [19], the place of downstream beds and rehabilitation units need more assessment [20]. We acknowledge that the choice of a log-normal distribution and the small number of young patients who died led to more uncertainty in the duration estimations. However, the sensitivity analysis using shorter tailed distribution was consistent with our main analysis.

Methodological issues in all analyses included the need to work with censoring (5%). This made it more difficult to conclude on the eventual portion of each outcome as some patients were still hospitalized at the time of the analysis. A number of early reports regarding patients’ outcomes did not consider these issues and likely underestimated the length of stay [21]. Using mixture for survival analysis has several advantages in this respect [22]. By comparison with competing risk models, mixture models posit the existence of several subpopulations, each submitted to one risk (death or survival) rather than one population submitted to several risks (death and survival) and allows estimating the importance of these subpopulations.

The overall mortality rate was 20%, with strong variation depending on age. This is in accordance with other research where older male patients were reported to be more likely to die [23,24,25]. Importantly, we found that overall mortality had substantially decreased over time, all the more when differences in case-mix were taken into account. This suggests that patient management improved over time. While we do not have data to confirm it, those of us who treated the patients resorted more frequently to anticoagulation drugs and corticosteroids over time, as well as to high-flow oxygen therapy. These drugs and procedures have been linked to better outcomes for the patients [11,26,27] but further investigation of this issue would be required in our study.

We lack follow-up data for patients still hospitalized at the time of the study, and of those discharged to long-term care or to retirement homes. Identifying long-term sequelae is of major interest and requires a prolonged and standardized follow-up, as recent work demonstrated that the symptoms of COVID-19 may persist beyond two months after onset [28].

## 5. Conclusions

COVID-19 patient hospitalization trajectories depended on age. The high proportion of ICU stays, and the long length of stay, make this disease challenging in terms of hospital management. Reducing ICU entrance by improving initial management and reducing the length of stay in ICU and in conventional departments remain the main challenges.

## Figures and Tables

**Table 1:** Characteristics of patients hospitalized for COVID-19 in the participating hospitals. Data show median [IQR] or percentage (number). IQR=Interquartile Range. ICU=Intensive Care Unit.

Characteristics	Age Group (*n*)			Overall (*n* = 1321)
	18–65 (*n* = 523)	66–80 (*n* = 400)	>80 (*n* = 398)	
Male (% (*n*))	57% (300)	60% (232)	44% (168)	55% (700)
Age (years) (median [IQR])	52.0 [43.9, 59.0]	72.0 [68.0, 75.8]	86.0 [83.0, 90.0]	69.0 [55.8, 82.0]
Time from onset to hospitalization (days) (median [IQR])	8 [5, 11]	7 [3, 10]	4 [2, 8]	7 [3, 10]
ICU < 24 h after admission (% (*n*))	17% (91)	20% (78)	6% (23)	14% (192)

**Table 2:** Characteristics of COVID-19 patient trajectories in the hospital according to age. All durations are median [IQR] for the lognormal distributions, corrected for censoring. IQR = Interquartile Range.

	Age			
Trajectory Characteristics	18–65	66–80	>80	Overall
	(*n* = 523)	(*n* = 400)	(*n* = 398)	(*n* = 1321)
Overall length of stay	10 [6, 16]	14 [9, 24]	13 [8, 21]	12 [7, 20]
Death (%)	9	20	38	20
Time to death (days)	53 [31, 91]	16 [9, 28]	10 [6, 17]	14 [8, 25]
Time to discharge (days)	8 [5, 13]	14 [9, 22]	14 [9, 23]	12 [7, 19]
ICU (%)	30	34	7	24
Time to ICU (days)	1 [0, 2]	1 [0, 3]	1 [0, 3]	1 [0, 2]
Time in the ICU (days)	20 [10, 39]	22 [11, 42]	9 [5, 18]	20 [10, 39]
Death in the ICU (%)	18	42	62	33
Time to death (days)	40 [22, 75]	23 [13, 44]	9 [5, 16]	27 [14, 52]
Discharge from the ICU (%)	82	58	38	67
Time to discharge (days)	17 [9, 34]	20.0 [10, 38]	10 [5, 19]	17 [9, 33]
No ICU (%)	70	66	93	75
Time in the hospital (days)	6 [4, 10]	9 [6, 15]	11 [7, 17]	9 [6, 14]
Death in hospital (%)	3	11	36	13
Time to death (days)	41 [25, 67]	8 [5, 12]	9 [6, 15]	9 [6, 15]
Discharge from hospital (%)	97	89	64	63
Time to discharge (days)	6 [4, 9]	10 [7, 15]	13 [9, 20]	9 [6, 14]

**Table 3:** Characteristics of patients hospitalized for COVID-19 in the participating hospitals by period of admission. Data show as median [IQR] or percentage (number).

	Period (*n*)			
	>15 March (*n* = 158)	15–30 March (*n* = 646)	1–15 April (*n* = 370)	1–15 April (*n* = 147)
Time from onset to hospitalization (days) (median [IQR])	5 [2, 9]	6 [3, 9]	6 [2, 10]	4 [2, 8]
ICU < 24 h after admission (% (*n*))	20 (31)	25 (159)	24 (89)	13 (19)
Length of stay (median [IQR])	13 [8, 21]	11 [7, 18]	12 [7, 20]	17 [10, 27]
Death (%)	22%	21%	17%	12%
Time to discharge (median [IQR])	13 [8, 21]	10 [6, 17]	12 [7, 19]	19 [12, 32]
Time to death (median [IQR])	13 [8, 21]	14 [8, 24]	13 [8, 22]	8 [5, 12]

## Supplementary Materials

The following are available online at https://www.mdpi.com/2077-0383/9/10/3148/s1: Detail of the mixture model. Sensitivity analysis, likelihoods for the trajectories models. Statistical Methods. Table S1: Characteristics of trajectories using exponential distributions; Table S2: log-likelihood of exponential and log-normal models.
