# Supplementary material for: Trajectories of Hospitalization in COVID-19 Patients: An Observational Study in France
Source: J Clin Med. 2020 Sep 29;9(10):3148. doi: 10.3390/jcm9103148 (PMC7600846; doi:10.3390/jcm9103148)
Supplement: Supplementary file 1 [file jcm-09-03148-s001.pdf]

Supplementary Materials:

Statistical Methods

Mixture Model

We fit models on patient trajectories recorded before and after hospital admission. Data consisted of age, sex, date of symptoms onset, and date of hospital admission, and subsequent dates of discharge or death, and, when relevant, dates of entering/leaving the ICU. We fit mixtures models to time-to-event data, taking into account censoring due to patients being still in the hospital at the time of analysis.

First, we modelled time to admission as a mixture of an exponential distribution and a lognormal distribution. The likelihood of an observed duration, d_i, was:

$$LL_i(\pi, \theta; d_i) = \pi \frac{F_\theta(d_i + 0.5) - F_\theta(d_i - 0.5)}{F_\theta(\min(T - d_i, 20) + 0.5)} + (1 - \pi) \frac{G_\theta(d_i + 0.5) - G_\theta(d_i - 0.5)}{G_\theta(\min(T - d_i, 20) + 0.5)}$$

where F and G were the exponential and lognormal distributions with parameter θ and mixture proportion π.

Then, we modelled time from admission to entering the ICU or being discharged/dead for those who do not go to the ICU. Write T for the time to the first of the 3 following events: entering the ICU, being discharged alive, or dying in the hospital. T is modelled as a mixture of 3 exponential distributions: T ~ $\pi_{ICU} Exp(\lambda_{ICU}) + \pi_{Dis} Exp(\lambda_{Dis}) + \pi_{Dth} Exp(\lambda_{Dth})$, where π_X is the probability to go to X (ICU, Discharge, or Death without ICU), and λ are the rates of the exponential distributions. The average time spent before the outcome is $1/\lambda_X$. The likelihood of a patient trajectory observed up to time t with final status s (comprising still hospitalized—HOS, admitted to ICU—ICU, discharged alive—DIS, dead—DTH) is given by:

$$L(\pi_{ICU}, \lambda_{ICU}, \lambda_{DIS}, \lambda_{DTH}) = (\pi_{ICU} \lambda_{ICU} \exp(-\lambda_{ICU}\, t))^{s=ICU}$$

$$(\pi_{DIS}\, \lambda_{DIS} \exp(-\lambda_{DIS}\, t))^{s=DIS} (\pi_{DTH}\, \lambda_{DTH} \exp(-\lambda_{DTH}\, t))^{s=DTH}$$

$$(1 - \pi_{ICU} \exp(-\lambda_{ICU}\, t) - \pi_{DIS} \exp(-\lambda_{DIS}\, t) - \pi_{DTH} \exp(-\lambda_{DTH}\, t))^{s=HOS}$$

The first line is for patients going to the ICU, the second line for those being discharged alive or dead, and the third line for patients who were censored because they were still in the hospital.

Likewise, we fit time to discharge or death after admission to the ICU using a mixture approach with mixture parameters ϕ and exponential parameters μ for being discharged alive or dead. The likelihood is therefore:

$$L(\mu_{DIS}, \mu_{DTH}) =$$

$$(\varphi_{DIS}\, \mu_{DIS} \exp(-\mu_{DIS}\, t))^{s=DIS} (\varphi_{DTH}\, \mu_{DTH} \exp(-\mu_{DTH}\, t))^{s=DTH})$$

$$(1 - \varphi_{DIS} \exp(-\mu_{DIS}\, t) - \varphi_{DTH} \exp(-\mu_{DTH}\, t))^{s=HOS}$$

As the data is rounded to the nearest day, we discretized the exponential distributions in the likelihood. All models were fitted at maximum likelihood using R.

For the overall analysis, we used:

$$L(\mu_{DIS}, \mu_{DTH}) =$$

$$(\varphi_{DIS}\,\mu_{DIS}\exp(-\mu_{DIS}\,t))^{s=DIS}\,(\varphi_{DTH}\,\mu_{DTH}\exp(-\mu_{DTH}\,t))^{s=DTH})$$

$$(1-\varphi_{DIS}\exp(-\mu_{DIS}\,t)-\varphi_{DTH}\exp(-\mu_{DTH}\,t))^{s=HOS}$$

to analyze the overall time in the hospital, irrespective of actual trajectory and parametrized φ_{DIS} to account for age, delay, and sex effect using a linear predictor on the logit scale.

Sensitivity Analysis

We fitted the models with exponential distributions rather than lognormal distributions. The results were very consistent with those reported in the manuscript.

Table S1: Characteristics of COVID-19 patient trajectories in the hospital according to age with exponential distributions, corrected for censoring. LOS: Length of Stay. Data are means.

	Age			
**Characteristics**	**18–65** (n = 523)	**66–80** (n = 400)	**>80** (n = 398)	**Overall** (n = 1321)
Overall LOS (days)	12.8	17.0	14.2	14.5
Death (%)	10.2	19.8	36.5	20.8
Time to death (days)	83.9	18.6	16.6	18.9
Time to discharge (days)	9.4	16.6	17.2	13.4
Time to ICU (days)	1.2	1.8	2.5	1.6
ICU (%)	30.0	33.8	6.6	24.1
Time in the ICU (days)	22.3	22.6	10.5	21.2
Death in the ICU (%)	26.7	47.0	60.7	38.2
Time to death (days)	71.5	31.3	8.3	34.6
Discharge from the ICU (%)	13.3	53.0	39.3	61.8
Time to discharge (days)	14.3	16.5	14.3	15.3
No ICU (%)	70.0	66.2	93.4	75.9
Time in the hospital (days)	7.8	11.0	12.6	10.4
Death in hospital (%)	3.9	10.7	34.7	16.6
Time to death (days)	73.9	8.8	9.0	10.9
Discharge from hospital (%)	96.1	89.3	65.3	83.4
Time to discharge (days)	6.5	11.9	16.5	10.9

Table S2: Log-likelihood for the trajectory models. This table shows the log-likelihood of the different models used for analysis. In summary, all models, including age, improved fit to the data. Models using a log normal distribution were also better than those using exponential distributions.

	**Age**		**All**	
Characteristics	Exponential	Log-normal	Exponential	Log-normal
Overall Exit	−4309.6	−4246.7	−4317.6	−4262.3
Overall Discharge/Death	−4756.5	−4716.1	−4889.9	−4834.1
ICU + exit	−4470.5	−4370.4	−4557.3	−4454.3
ICU + Death + Discharge	−4655.0	−4549.9	−4867.6	−4749.1
InICU: exit	−938.9	−934.3	−943.9	−938.1
InICU: death + discharge	−1059.1	−1058.7	−1083.4	−1079.7
